# The Association of Genetic Markers for Type 2 Diabetes with Prediabetic Status - Cross-Sectional Data of a Diabetes Prevention Trial

**DOI:** 10.1371/journal.pone.0075807

**Published:** 2013-09-30

**Authors:** Birgit-Christiane Zyriax, Ramona Salazar, Wolfgang Hoeppner, Eik Vettorazzi, Christian Herder, Eberhard Windler

**Affiliations:** 1 University Medical Center Hamburg-Eppendorf, Endocrinology and Metabolism of Ageing, Hamburg, Germany; 2 Bioglobe GmbH, Medical Genetics, Hamburg, Germany; 3 University Medical Center Hamburg-Eppendorf, Department of Medical Biometry and Epidemiology, Hamburg, Germany; 4 Institute for Clinical Diabetology, German Diabetes Center, Leibniz Center for Diabetes Research at Heinrich Heine University Düsseldorf, Düsseldorf, Germany; University of Bremen, Germany

## Abstract

**Objective:**

To investigate the association of risk alleles for type 2 diabetes with prediabetes accounting for age, anthropometry, inflammatory markers and lifestyle habits.

**Design:**

Cross-sectional study of 129 men and 157 women of medium-sized companies in northern Germany in the Delay of Impaired Glucose Tolerance by a Healthy Lifestyle Trial (DELIGHT).

**Methods:**

Besides established risk factors, 41 single nucleotide polymorphisms (SNPs) that have previously been found to be associated with type 2 diabetes were analyzed. As a nonparametric test a random forest approach was used that allows processing of a large number of predictors. Variables with the highest impact were entered into a multivariate logistic regression model to estimate their association with prediabetes.

**Results:**

Individuals with prediabetes were characterized by a slightly, but significantly higher number of type 2 diabetes risk alleles (42.5±4.1 vs. 41.3±4.1, p = 0.013). After adjustment for age and waist circumference 6 SNPs with the highest impact in the random forest analysis were associated with risk for prediabetes in a logistic regression model. At least 5 of these SNPs were positively related to prediabetic status (odds ratio for prediabetes 1.57 per allele (Cl 1.21–2.10, p = 0.001)).

**Conclusions:**

This explorative analysis of data of DELIGHT demonstrates that at least 6 out of 41 genetic variants characteristic of individuals with type 2 diabetes may also be associated with prediabetes. Accumulation of these risk alleles may markedly increase the risk for prediabetes. However, prospective studies are required to corroborate these findings and to demonstrate the predictive value of these genetic variants for the risk to develop prediabetes.

## Introduction

The prevalence of type 2 diabetes is dramatically increasing and represents a worldwide growing health problem [1]–[4]. Even in those individuals with prediabetes the risk for cardiovascular disease and total mortality is almost doubled [5]–[10]. A prediabetic status is also associated with microvascular complications [11]. Finally, 5 to 10% of untreated prediabetic patients will develop diabetes each year [12], [13]. Yet, the same proportion may convert back from the prediabetic status to normoglycemia [11]. In routine medical practice, prediabetes is not yet recognized nor treated, although it has been repeatedly demonstrated that the transition to type 2 diabetes can be delayed or avoided [14]. A sedentary lifestyle and an unhealthy dietary pattern promote weight gain, particularly central adiposity, which increases the risk for prediabetes and eventually type 2 diabetes [12], [13]. Adipocytokines such as leptin and adiponectin or the proinflammatory cytokine interleukin-6 (IL-6) are affected by lifestyle habits and seem to play an important role for weight development, body composition and risk for type 2 diabetes [15]–[20]. In addition, a family history of type 2 diabetes markedly increases the risk for diabetes reflecting the interaction of genetic factors with modern lifestyle and anthropometry [21], [22]. Results from family studies and research in various ethnic groups indicate that the heritability of the disease may exceed 50% [23]. Still, up to now information on the impact of multiple gene loci as to the risk for prediabetes is limited. In a cohort of non-diabetic Caucasians a significant association between impaired glucose tolerance and risk alleles for type 2 diabetes has been shown for female and obese individuals, whereas it has not been possible to demonstrate an effect in male, lean and insulin sensitive subjects [24].

Until 2011 approximately 40 diabetes-associated genes had been identified [23], [25]–[28]. Single nucleotide polymorphisms (SNPs) are the most commonly investigated type of specific genetic variants. However, the identification of a single gene variant associated with a complex disease such as diabetes among a large number of SNPs by statistical methods such as logistic regression analysis has limitations [29]–[31]. As more SNPs and interaction terms are added, the model becomes unstable in the sense that the variance of the parameter estimates becomes excessively large or even inestimable, when the number of model parameters exceeds the number of cases, and the effect of a genetic variant can be neutralized by the interaction with related parameters. Lately, new nonparametric predictive models have been developed to overcome this problem such as the random forest analysis, which attracts growing interest. One major advantage of this statistical approach is its capability to cope with a large number of predictors and to identify those factors with a relevant contribution to the disease, even in the presence of high order interactions [31]–[33].

This prompted us to analyze cross sectional data of young employees in the Delay of Impaired Glucose Tolerance by a Healthy Lifestyle Trial (DELIGHT) as to the association of 41 SNPs indicating risk for type 2 diabetes with a prediabetic status [23], [25]. This research uses random forest analysis to identify genetic markers of prediabetes that may add to the information of anthropometric data, inflammatory markers and lifestyle factors as to the risk for developing prediabetes [31]–[33].

## Methods

### Ethics Statement

The study protocol was approved by the ethical committee of Hamburg and conducted according to the principles of the Declaration of Helsinki. Written informed consent was obtained from all participants. The trial was registered in the German Clinical Trials Register No. DRKS00000695 (www.germanctr.de).

### Design and Recruitment

DELIGHT is a feasibility study on sustainable prevention of diabetes in young men and women. 18–65 year-old employees of 5 medium-sized companies in the northern part of Germany were informed about prediabetes, risk for diabetes, and chance of lifestyle modification [34]. Employees were advised how to measure their waist circumference, and were eligible for a check-up, if the waist circumference was ≥80 cm for women and ≥94 cm for men or close to these cut-off points.

Exclusion criteria were known pregnancy, known type 1 or type 2 diabetes, or acute malignant or severe chronic diseases. The final study population comprised 300 participants. However, the present analysis focuses on the data at screening of 129 men and 157 women for whom complete information about lifestyle habits, anthropometric parameters, laboratory values and genetic data were available.

### Data Collection

#### Assessment of anthropometric data and lifestyle

Height and weight - light clothing, but no shoes allowed - were measured to the nearest 0.5 cm or 0.1 kg, respectively, and body mass index (BMI) was calculated as BMI = (weight, kg)/(height, m)^2^. Waist circumference was measured in the middle between the lower rib margin and the iliac crest. Central obesity was defined by a waist circumference ≥80 cm in women and ≥94 cm in men [35].

Information on lifestyle, nutrition, socio-demographic characteristics and family history of diabetes was obtained using validated questionnaires developed for the EPIC study (European Prospective Investigation into Cancer and Nutrition), a prospective multicenter cohort study in Europe, investigating the association between lifestyle factors and chronic diseases [36]–[38]. A self-administered food questionnaire recorded the frequency and portion size of 146 food items eaten during the preceding year. Physical activity was calculated as sports in hours per week, taken into account activities during summer and wintertime. Smoking habits were described as number of cigarettes per day.

### Laboratory and Clinical Data

Plasma fasting glucose and plasma glucose two hours after oral challenge with 75 g glucose (oral glucose tolerance test - OGTT) were measured from Na-fluoride-containing Monovettes (Sarstedt AG & Co, Nümbrecht, Germany). Routine laboratory parameters were determined by standard techniques in the central laboratory of the University Medical Center Hamburg-Eppendorf. Low-density lipoprotein (LDL) -cholesterol using the Friedewald formula. Prediabetes was defined as fasting blood glucose levels (IFG) between 100–<126 mg and/or plasma glucose levels two hours after an oral load of 75 g glucose (IGT) between 140–199 mg/dl. Diabetes was defined as fasting plasma glucose levels ≥126 mg/dl and/or ≥200 mg/dl two hours after 75 g of glucose [10].

Serum concentrations of IL-6 and adiponectin were measured using the Quantikine HS ELISA kit and the Quantinkine ELISA kit, respectively (R&D Systems, Wiesbaden, Germany) as described [38]. Serum leptin concentrations were determined with a bead-based assay using a Luminex 100 analyser (Luminex Corporation, Austin, TX, USA) as described [39].

Blood pressure was taken in a sitting position 3 times approximately 2 min. apart, of which the second and third value were averaged [40]. Hypertension was defined by antihypertensive medication or blood pressure ≥140 mmHg/≥90 mmHg. The homeostasis model assessment insulin resistance (HOMA-IR) score was categorized at 2.5 as the suggested upper limit of normal and ≥3.8, the upper quartile of a European population [41]–[42]. For the definition of the metabolic syndrome the criteria of the International Diabetes Federation were adopted [43].

### Genetic Data

DNA was isolated from blood samples using the QIAamp DNA blood Mini Kit (Qiagen, Hilden, Germany). The gene polymorphisms of 41 identified SNPs for the risk of type 2 diabetes (Table 1) with a minor allele frequency of at least 1% in a population of European descent were analyzed by matrix assisted laserdesorption/ionization time-of-flight mass spectrometry (MALDI-TOF MS) using the Sequenom MassARRAY platform (Sequenom, San Diego, CA, USA). Assay design was performed using the standard design procedure supported by the system supplier (www.mysequenom.com). Primers were synthesized by Metabion, Martinsried, Germany and Biomers, Ulm, Germany. iPLEX GOLD application was carried out according to manufacturer’s instructions and as described previously [44]. Routinely 5% of samples were randomly picked for duplicate genotyping. The concordance was 100%.

**Table 1:** Gene loci and SNPs associated with increased risk of type 2 diabetes.

Gene locus	Cytogenetic location	Gene name	SNP
ADAMTS9	3p14.3-p14.2	ADAMTS9 antisense RNA 2	rs4607103
ADCY5	3q13.2-q21	adenylate cyclase 5	rs11708067
BCL11A	2p16.1	B-cell CLL/lymphoma 11A	rs243021
C2CD4B	15q21.3	C2 calcium-dependent domain containing 4A/B	rs7172432
CDC123	10p13-p14	cell division cycle 123	rs12779790
CDKAL1	6p22.3	CDK5 regulatory subunit associated protein 1-like 1	rs7754840
CDKN2AB	9p21.3	cyclin-dependent kinase inhibitor 2A/2B	rs10811661
CENTD2	11q13.4	Arf-GAP with RhoGAP domain, ankyrin repeat and PH domain 1	rs1552224
CHCHD9	9q21.31	coiled-coil-helix-coiled-coil-helix domain containing 9	rs13292136
DGKB	7p21.2	diacylglycerol kinase, beta 90 kDa	rs2191349
DUSP9	Xq28	dual specificity phosphatase 9	rs5945326
FTO	16q12.2	fat mass and obesity associated	rs8050136
FTO	16q12.2	fat mass and obesity associated	rs9939609
GCK	7p15.3-p15.1	glucokinase	rs4607517
GCKR	2p23.3-p23.2	glucokinase (hexokinase 4) regulator	rs780094
HHEX	10q24	hematopoietically expressed homeobox	rs1111875
HMGA2	12q14.3	High mobility protein group HMCI-C	rs1531343
HNF1A	12q24.2	Hepatocyte nuclear factor 1-alpha	rs7957197
HNF1B	17q12	HNF1 homeobox B	rs4430796
IGF2BP2	2q33-q34	insulin-like growth factor 2 mRNA binding protein 2	rs1470579
IGF2BP2	2q33-q34	insulin-like growth factor 2 mRNA binding protein 2	rs4402960
IRS1	2q36	insulin receptor substrate 1	rs2943641
JAZF1	7p15	JAZF zinc finger 1	rs864745
KCNJ11	11p15.1	potassium inwardly-rectifying channel, subfamily J, member 11	rs5219
KCNQ1	11p15.5	potassium voltage-gated channel, KQT-like subfamily, member 1	rs231362
KLF14	7q32.3	Kruppel-like factor 14	rs972283
MTNR1B	11q21-q22	melatonin receptor 1B	rs10830963
NOTCH2	1p13-p11	notch 2	rs10923931
PPARG	3p25	peroxisome proliferator-activated receptor gamma	rs1801282
PRC1	15q26.1	protein regulator of cytokinesis 1	rs8042680
PROX1	1q32.2-q32.3	prospero-related homeobox 1	rs340874
RBMS1	2q24.2	RNA binding motif, single stranded interacting protein 1	rs7593730
SLC30A8	8q24.11	solute carrier family 30 (zinc transporter), member 8	rs13266634
TCF7L2	10q25.2-q25.3	transcription factor 7-like 2	rs7903146
THADA	2p21	thyroid adenoma associated	rs7578597
TP53INP1	8q22.1	tumor protein p53 inducible nuclear protein 1	rs896854
TSPAN8	12q21.1	tetraspanin 8	rs7961581
UBE2E2	3p24.3	ubiquitin-conjugating enzyme E2E 2	rs7612463
WFS1	4p16.1	Wolfram syndrome 1	rs10010131
ZBED3	5q13.3	ZBED3 antisense RNA 1	rs4457053
ZFAND6	15q25.1	AN1-type zinc finger protein	rs11634397

### Statistical Analyses

Baseline characteristics of the participants were reported as means and standard deviation for quantitative data and are compared between groups using Student’s t-test or Oneway ANOVA, depending on the number of groups. Qualitative scales are reported as counts and proportions and compared using chi-squared tests. Selected quantitative data like waist or BMI, HOMA-IR were also reported using discretized versions with clinically defined cut points. P-values below 0.05 were considered statistically significant. The random forests approach, a collection of classification trees, was used to cope with the large number of variables and select those markers with a relevant contribution to the defined outcome variable ‘prediabetic status’. The model of the random forests approach has been described previously in detail [32], [33]. Statistical calculations were performed running the software version R2.15.1 using the forest procedure from the party package [45]–[48]. The most promising variables found in this analysis were used to set up a multivariate logistic regression model to estimate the predictive value of identified parameters individually and collectively.

## Results

### Baseline Characteristics

About one third of the study population, men and women likewise, were affected by prediabetes, identified by elevated fasting and/or 2-h glucose (Table 2). Mean age and body mass index (BMI) did not differ between men and women (data not shown). However, women were characterized more often by an elevated waist circumference, and by higher levels of leptin and adiponectin, but also by a more favorable lipid profile. The prevalences of hypertension and prediabetes were comparable in both sexes. As to dietary and lifestyle habits women were characterized by a lower intake of energy, fat, saturated fat and fiber than men. Smoking habits did not differ between sexes, whereas females reported less physical activity.

**Table 2:** Baseline characteristics of normoglycemic versus prediabetic participants.

Clinical characteristics	Subcategory	Normoglycemic n = 197(68.9%)	Prediabetic n = 89(31.1%)	p-value
Sex	Male [%]	69.8	30.3	n.s.†
	Female [%]	68.2	31.8	
Age [years]		42.6±8.7	47.3±8.2	<0.001§
BMI [kg/m^2^]		28.1±4.4	30.2±5.1	<0.001§
	<25 [%]	23.9	13.5	0.008†
	25–<30 [%]	49.7	42.7	
	≥30 [%]	26.4	43.8	
Waist circumference [cm]		92.8±11.4	98.5±12.7	<0.001§
	<94 cm*/<80 cm^#^ [%]	23.5	10.1	0.002†
	≥94 cm*/≥80 cm^#^ [%]	37.2	30.3	
	≥102 cm*/≥88 cm^#^ [%]	39.3	59.6	
Fasting glucose [mg/dl]		91.0±5.1	104.9±5.2	<0.001§
	≥100 mg/dl [%]	0	96.6	<0.001†
	<100 mg/dl [%]	100	3.4	
2-h glucose [mg/dl]		81.6±20.3	101.7±29.3	<0.001§
	<140 mg/dl [%]	0	13.5	<0.001†
	≥140 mg/dl [%]	100	86.5	
HOMA-IR		2.0±4.2	2.7±2.7	n.s.§
	<2.5 [%]	83.6	61.4	<0.001†
	2.5–<3.8 [%]	7.7	19.3	
	≥3.8 [%]	8.7	19.3	
Triglycerides ([mg/dl]		122.4±68.8	163.1±134.4	<0.001§
HDL-cholesterol [mg/dl]		62.1±16.4	60.0±16.5	n.s.§
LDL-cholesterol [mg/dl]‡		123.6±33.1	127.8±32.5	n.s.§
Hypertension [%]		20.0	42.5	<0.001†

Values are given as mean ±1 standard deviation or as absolute or relative frequencies.

* males / ^#^females;

§ t-test,

† chi-square test;

‡ estimated by the Friedewald formula.

Participants with prediabetes were older, had a higher HOMA-IR, and a higher BMI particularly within the category of obesity compared with normoglycemic subjects (Table 2). Also, triglyceride levels and the rate of hypertension were significantly higher in prediabetic individuals, whereas plasma concentrations of LDL- and HDL-cholesterol were similar. No differences were observed as to mean values of leptin, adiponectin, and IL-6, but also dietary intake and physical activity (data not shown).

### Genetic Data

Within the study population the total number of risk alleles did not differ between men and women (Table 3). Individuals with prediabetes were characterized by a slightly, but significantly higher number of total risk alleles (Table 3). Categories of HOMA-IR did not differ in the number of risk alleles, but trends towards higher numbers of risk alleles for increasing categories of fasting glucose levels and 2-h glucose levels were observed (Table 3).

**Table 3:** Number of total homozygous or heterozygous risk alleles.

Genetic data		Number ofrisk alleles	p-value
Sex	Male	41.6±3.9	n.s.
	Female	41.8±3.7	
Normoglycemic		41.3±3.6	p = 0.013
Prediabetic		42.5±4.1	
HOMA-IR	<2.5	41.7±3.9	p = 0.738
	2.5–<3.8	42.2±3.1	
	≥3.8	41.5±3.6	
Fasting glucose[mg/dl]	<90 mg/dl	41.1±3.8	p = 0.059
	90–<100 mg/dl	41.5±3.6	
	≥100 mg/dl	42.5±4.1	
2-h glucose [mg/dl]	<140 mg/dl	41.6±3.8	p = 0.128
	≥140 mg/dl	43.3±4.6	

Values are given as mean ±1 standard deviation.

To identify genetic markers with a greater contribution as to risk for prediabetes the random forest approach was used. 41 SNPs for which associations with type 2 diabetes were published until 2011, and as well age, sex, anthropometric data, inflammatory markers (leptin, adiponectin, IL-6) and lifestyle factors known to contribute to diabetes (total energy intake, fat intake, intake of saturated fat and fiber) were included in the model.

**Figure 1:**
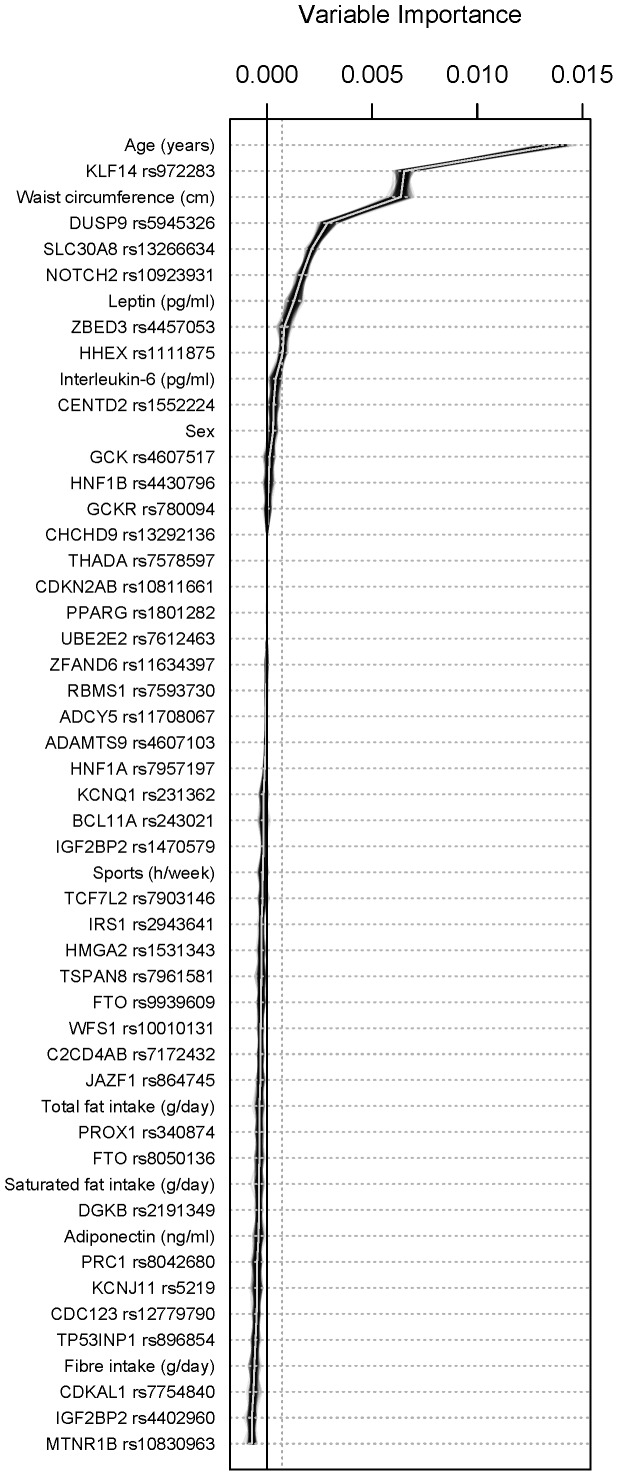
Relevance of markers as to prediabetic status of 100 runs in a random forest analysis.

In this explorative study those markers that exceeded the random fluctuation around zero - the magnitude of which is indicated by the negative variation and the dotted line in Figure 1 - were selected for further analyses as suggested by [49]. Markers of relevance comprised age, waist circumference, and leptin, but also 6 SNPs: rs972283 in *KLF14*, rs5945326 in *DUSP9*, rs13266634 in *SLC30A8*, rs10923931 in *NOTCH2*, rs4457053 in *ZBED3*, and rs1111875 in *HHEX* (Figure 1).

**Figure 2:**
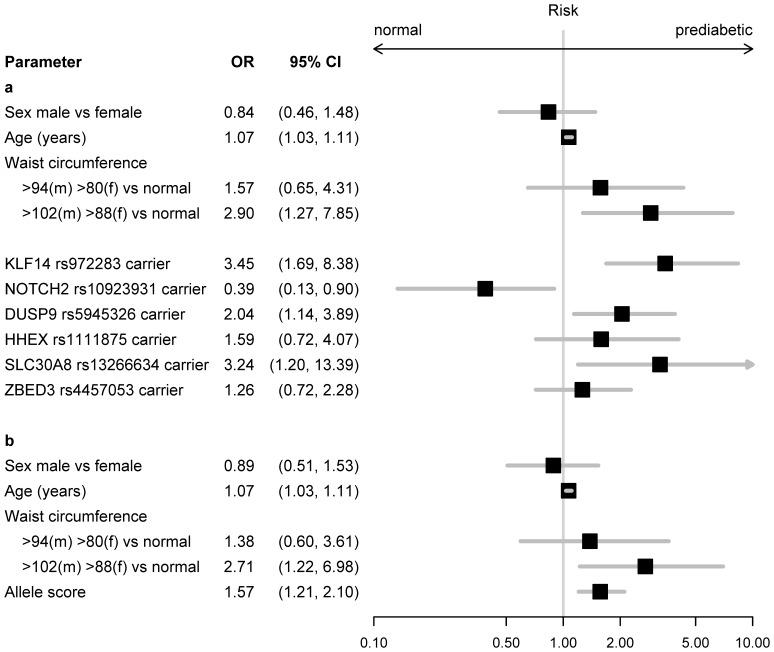
Logistic regression as to risk for prediabetes of SNPs per allele (2a) or sum score (2b).

According to the analysis obtained by the random forest model the 6 SNPs representing the most powerful genetic markers were selected. Since the random forest approach does not distinguish whether the identified SNP may increase or decrease susceptibility for the disease, a logistic regression was performed including age, sex, categories of waist circumference and the 6 selected SNPs (Figure 2a). The results indicate that sex was not associated with increased risk for prediabetic status, whereas age and central obesity, particularly a waist circumference ≥88 cm in women and ≥102 cm in men, were significantly related to a higher risk. The majority of SNPs showed a tendency towards a higher risk as to prediabetic status, which was significant in carriers of rs972283 in *KLF14*, rs5945326 in *DUSP9*, and rs13266634 in *SLC30A8* (Figure 2a). However, rs10923931 in *NOTCH2* was significantly associated with a lower risk as to prediabetic status. To calculate the effect per risk allele, in the next step a logistic regression was performed including age, sex, categories of waist circumference and the 6 identified SNPs as sum score (Figure 2b). With every SNP the odds for prediabetes increased by 57% (Cl 1.21–2.10, p = 0.001). Evaluation as to hetero- and homozygote carriers showed similar results (data not shown). Exclusion of rs10923931 in *NOTCH2* would lead to 92% (Cl 1.43–2.68, p<0.0001) increase in risk per allele. Including leptin in the analysis did not change the results (leptin 1.01 Cl 0.97–1.05; allele score 1.93 Cl 1.44–2.72).

## Discussion

Compared to type 2 diabetes, the information on the impact of multiple gene loci as to the risk for prediabetes is limited and needs further clarification. Analysis of the DELIGHT data indicates that genetic variants, which predispose individuals to type 2 diabetes, may serve as risk markers for the development of prediabetes as well. Individuals with prediabetes were characterized by a significantly higher number of risk alleles than normoglycemic subjects. On average each relevant SNP increased the odds for prediabetes by 57%. Accumulation of these risk alleles may lead to a markedly increase of the risk for prediabetes, the extent of which certainly needs to be determined in adequately sized prospective studies.

There is strong evidence that a prediabetic status is sufficient to increase the risk of cardiovascular disease and death substantially [5]–[10]. Notably, in DELIGHT one third of the relatively young and healthy employees pre-selected by an elevated waist circumference was affected by prediabetes, most of them as part of a metabolic syndrome. Once identified, successful lifestyle intervention trials clearly show that diabetes may be delayed, if not prevented. Untreated, 5 to 10% prediabetic patients may develop diabetes each year [12], [13]. Therefore early detection of individuals at risk is a major challenge. Obviously, genetic markers can be determined early in life, an advantage compared to established risk factors, which confer an elevated risk primarily at a later stage. In DELIGHT the impact of well-established risk factors such as lifestyle habits or inflammatory markers appeared to be rather small, possibly explained by an elevated waist circumference as an inclusion criterion. Since age and anthropometry though risk factors for prediabetes, lack specificity, an array of simple genetic markers may be helpful to identify individuals at risk.

In the present analysis a slightly, but significantly higher number of total risk alleles characterized individuals with prediabetes compared to normoglycemic subjects. This finding was not sex-linked. In the TUEbingen Family study (TUEF) Lindner et al. reported that genetic risk alleles predict risk for impaired glucose tolerance [24]. This was only shown for women and obese individuals, yet. However, at that time the results were based on only 9 selected diabetes-associated genes, particularly those related to impaired glucose tolerance. Differences as to the influence of sexes may be explained by an underrepresentation of males in the TUEbingen Family study. This is supported by some studies which found that sex-hormones differently modulate glycemic status and IGT is more frequent in males, whereas IFG occurs more often in females [50]–[51].

Observational studies indicate that both parameters, elevated fasting and 2-h glucose values after an OGTT, seem to be strong predictors of diabetes incidence [52]–[59]. However, one should properly distinguish between variants obtained from genome-wide studies focusing on type 2 diabetes and those genes examined in epidemiological studies that are responsible for the regulation of glucose levels within the normal range [23], [60]. In DELIGHT clinically established categories of IFG and IGT showed a tendency towards a higher number of risk alleles. Yet, the risk of diabetes may be higher in subjects with isolated IGT compared to those with isolated IFG [55]. Pathophysiological mechanisms of isolated IFG and isolated IGT probably differ, but the finding and its clinical relevance need further clarification [11]. The small proportion of individuals with isolated elevated IGT in the DELIGHT project may be a matter of both the inclusion criteria of an elevated waist circumference and a low threshold of 100 mg/dl for IFG in contrast to 110 mg/dl as formerly used.

Results from the random forest analysis indicate that particularly age and waist circumference, but also leptin and 6 single-nucleotide polymorphisms out of 41 are associated with an elevated risk for prediabetes. The impact of age, waist circumference and leptin is in line with other investigations on risk for diabetes. Interestingly, sex, adiponectin, interleukin-6 levels and lifestyle habits were not selected as markers with a particularly important contribution to the disease by the random forest model. As to adiponectin and lifestyle habits, one explanation might be that age, waist circumference, leptin levels and some of the risk alleles cover much of the risk common to the preselected study population [21], [61]–[66].

In a logistic regression model the majority of the 6 selected SNPs were positively associated with prediabetic status. However, a strong significant effect was only revealed in carriers of rs972283 in *KLF14*, rs5945326 in *DUSP9*, and rs13266634 in *SLC30A8*, explainable either by the smaller sample size of our study or pre-selection of the participants by waist circumference. Variations at *KLF14*, the Krueppel like factor 14, were related to type 2 diabetes and HDL-cholesterol but also basal cell carcinoma in different populations [24], [26], [67]–[73]. The effect of *KLF14* is reportedly not driven by obesity, quite unlike the known BMI- and fat mass mediated effect of *FTO* via insulin resistance [26], [74], [75]. Additionally, rs5945326 in *DUSP9*, the dual specificity protein phosphatase 9, and rs13266634 in *SLC30A8,* the zinc transporter, were positively related to prediabetic status. Results from other investigations indicate that DUSP9 and *SLC30A8* are common susceptibility loci for type 2 diabetes across various ethnicities [23], [25], [76]–[78]. Furthermore, a positive association between prediabetic status and *HHEX* and *ZBED* was revealed. These findings are supported by others who investigated the effect of the selected SNPs as to risk for type 2 diabetes [23], [26], [60], [79].

Pre-selection criteria as to central obesity within the DELIGHT project, the exclusion of participants with known type 2 diabetes, or the sample size may have biased our result. Therefore previously identified risk variants in other gene loci such as *TCF7L2* or *FTO* failed to show an important relationship in our analysis or were even associated with a decreased risk such as *NOTCH2*.

### Limitations and Strength of this Study

DELIGHT has limitations that need to be addressed. First, our findings are confined to those employees who voluntarily chose to take part in the program, and were characterized by central obesity or at least a waist circumference close to the threshold. Second, the sample size was rather small and therefore associations between several identified SNPs and prediabetic status may fail to reach statistical significance. However, DELIGHT is one of the first studies, to evaluate the association between a wide array of SNPs published at the time of this analysis and risk of prediabetes above and beyond established predictors. This was possible by applying the advanced statistical method of a random forest analysis. Advantage of this explorative approach is not only the capability of coping with large numbers of predictors even in the presence of complex interactions that may have any impact.

### Conclusions

This explorative analysis of DELIGHT demonstrates that at least 6 out of 41 genetic variants characteristic of individuals with type 2 diabetes may be related to prediabetic status as well. With every SNP the odds for prediabetes increased significantly beyond well-established risk factors such as age and waist circumference. In the future the identification of those markers may be useful in clinical practice to identify individuals at risk at an early stage. Certainly, more research using prospective data is required to confirm these findings, obtained by the application of the method of selected random forest analysis, to establish a clinically applicable tool.
